# Development of an occupational advice intervention for patients undergoing lower limb arthroplasty (the OPAL study)

**DOI:** 10.1186/s12913-018-3238-z

**Published:** 2018-06-28

**Authors:** Paul Baker, Carol Coole, Avril Drummond, Catriona McDaid, Sayeed Khan, Louise Thomson, Catherine Hewitt, Iain McNamara, David McDonald, Judith Fitch, Amar Rangan

**Affiliations:** 1 South Tees Hospitals NHS Foundation Trust, Middlesbrough, England UK; 2 The University of Nottingham, Nottingham, England UK; 3 York Trials Unit, Department of Health Sciences, The University of York, York, England UK; 4 Norfolk and Norwich University Hospital NHS Foundation Trust, Norwich, England UK; 5 University of Stirling, Stirling, Scotland UK; 6 British Orthopaedic Association Patient Liaison Group (BOA PLG), London, England UK; 7 Faculty of Medical Sciences, University of Oxford, Oxford, England UK

**Keywords:** Occupational advice, Return to work, Arthroplasty, Hip, Knee, Intervention design, Intervention mapping

## Abstract

**Background:**

There are an increasing number of patients of working age undergoing hip and knee replacements. Currently there is variation in the advice and support given about sickness absence, recovery to usual activities and return to work after these procedures. Earlier, sustainable, return to work improves the health of patients and benefits their employers and society. An intervention that encourages and supports early recovery to usual activities, including work, has the potential to reduce the health and socioeconomic burden of hip and knee replacements.

**Methods/design:**

A two-phase research programme delivered over 27 months will be used to develop and subsequently test the feasibility of an occupational advice intervention to facilitate return to work and usual activities in patients undergoing lower limb arthroplasty. The 2 phases will incorporate a six-stage intervention mapping process:

Phase 1: Intervention mapping stages 1–3:

1. Needs assessment (including rapid evidence synthesis, prospective cohort analysis and structured stakeholder interviews)
2. Identification of intended outcomes and performance objectives
3. Selection of theory-based methods and practical strategies

Phase 2: Intervention mapping stages 4–6:

4 Development of components and materials for the occupational advice intervention using a modified Delphi process
5 Adoption and implementation of the intervention
6 Evaluation and feasibility testing

The study will be undertaken in four National Health Service (NHS) hospitals in the United Kingdom and two Higher Education Institutions.

**Discussion:**

OPAL (Occupational advice for Patients undergoing Arthroplasty of the Lower limb) aims to develop an occupational advice intervention to support early recovery to usual activities including work, which is tailored to the requirements of patients undergoing hip and knee replacements. The developed intervention will then be assessed with a specific focus on evaluating its feasibility as a potential trial intervention to improve speed of recovery to usual activities including work.

**Trial registration:**

The study was registered retrospectively with the International Standard Randomised Controlled Trials Number (ISRCTN): 27426982 (Date 20/12/2016) and the International prospective register of systematic reviews (PROSPERO): CRD42016045235 (Date 04/08/2016).

## Background

Lower limb joint replacement is an effective and cost-effective way of relieving pain, restoring physical function and improving health related quality of life for patients with hip and knee arthritis. Currently over 170,000 hip and knee replacements are performed annually in England, Wales and Northern Ireland [1]. The decreased physical function associated with arthritis reduces the likelihood of employment, reduces household income and increases missed workdays for those who are employed [2]. This observation, combined with an ageing workforce and changes to the pension age, has resulted in a steady increase in the numbers of hip and knee replacements being performed in patients of working age over the last decade [1].

Currently 82% of people aged 35 to 49 and 67% aged 50 to 64 years are in paid work [3] with many more ‘working’ in unpaid roles as volunteers and carers. In 2015, 17,293 of 84,462 (20%) hip replacements and 16,121 of 94,437 (17%) knee replacements performed in England, Wales and Northern Ireland were in patients aged under 60 years; a further 25,249 (30%) hip replacements and 32,321 (34%) knee replacements were performed in patients aged between 60 and 69 years [1].

After hip and knee replacement 71–98% of patients return to work, although the mean time to return varies substantially (2–14 weeks) dependent upon the surgery performed, type of work undertaken and the return to work outcomes used [4]. An estimated 8.8 million working days were lost in 2015/16 due to work related musculoskeletal disorders, accounting for 34% of all working days lost due to work related ill health [5]. The costs associated with ill health preventing work are borne by the individual (impact of ill health on quality of life), employers and society (loss of productivity, sick pay, need for health care, rehabilitation and compensation). Lengthy sickness absence can result in work disability, poorer general health, increased risk of mental health problems and higher mortality [6]. Earlier sustained return to work therefore has potential health benefits as well as socioeconomic value.

### Research question and study title

The study is funded by the National Institute for Health Research (NIHR) Health Technology Assessment (HTA) programme in response to a commissioned call: ‘Occupational advice initiated prior to planned surgery for lower limb joint replacement’ (HTA Ref: 15/28/02). The research question is: ‘How feasible is a trial to evaluate whether an occupational advice intervention delivered to working adults, commencing prior to primary hip or knee joint replacement surgery, improves speed of recovery to usual activities including work?’. The working title and study acronym are: Occupational Advice for Patients undergoing Arthroplasty of the Lower limb (OPAL). The paper is based on version 4.0 of the OPAL protocol.

### Knowledge gaps

The interaction between patients, employers and surgical intervention is complex. Return to work is influenced by a range of patient, health process and employment factors [4]. The underlying probability of employment also varies by age, gender, education level, and other factors, meaning the economic implications of musculoskeletal limitations vary between patients and regions. If a clinical trial is to be undertaken, then a tailored occupational advice intervention must first be developed that considers these variations and the factors that influence the outcome of interest (recovery to usual activities including work). Unfortunately these factors are poorly understood and, as a result, there is significant variation in current practice and with the advice currently delivered to patients returning to work following their surgery. A number of specific gaps therefore require attention before an occupational advice intervention is ready to be evaluated in a randomised controlled trial.

Important considerations include:

- Defining the target population for a trial
- Current recommendations guiding return to work are limited and inconsistent. The provision and utility of occupational advice within ‘usual care’ pathways is not currently clear. There is therefore no appropriate occupational advice intervention available that could be used as the intervention arm in a trial.
- ‘Standard care’ is not currently defined for use as a study comparator
- The suitability of return to work measures as outcome tools for a trial is currently unknown

## Methods/design

The aim of the OPAL study is to develop an occupational advice intervention to support early recovery to usual activities including work, which is tailored to the requirements of patients undergoing hip and knee replacements, and to test the acceptability, practicality and feasibility of this intervention within current care frameworks. Based on the knowledge gaps within the current evidence the OPAL study has the following objectives:

1. To evaluate the specific needs of the population of patients who are in work and intend to return to work following hip and knee replacement.
2. To establish how individual patients return to work; the role of fit notes, clinical and workplace-based interventions, and how specific job demands influence workplace disability and productivity.
3. To establish what evidence is currently available relating to return to work / occupational advice interventions following elective surgical procedures.
4. To understand the barriers preventing return to work that need to be addressed by an occupational advice intervention.
5. To construct a multi-stakeholder intervention development group to inform the design and establish the necessary components of an evidenced based occupational advice intervention initiated prior to planned lower limb joint replacement.
6. To develop and manualise a multidisciplinary occupational advice intervention tailored to the needs of this patient group.
7. To determine current models of delivering occupational advice; the nature and extent of the advice offered; and how tools to facilitate return to work are being currently used.
8. To define a suitable measure of ‘return to work’ through systematic review and evaluation of specific measures of activity, social participation and return to work including specific validated workplace questionnaires.
9. To test the acceptability, practicality and feasibility and potential cost of delivering the manualised intervention within current care frameworks and as a potential trial intervention.

The stated objectives will be achieved in **2 phases** and be delivered over **27 months**:

- **Phase 1** will take place in the first 13 months and will address aims 1–4, 7 and 8 by gathering information on current practice and barriers to change; it will also provide a theoretical framework for intervention development.
- **Phase 2** will use information from phase 1 and provide the context for intervention development and testing. It will address aims 5, 6 and 9 and will be delivered in the final 17 months.

OPAL will use an intervention mapping approach that has been used previously to successfully develop and assess occupational advice interventions within musculoskeletal medicine [7] and other surgical specialties [8]. Intervention Mapping (IM) is a stepwise approach to theory, evidence based development and implementation of interventions [9, 10]. IM consists of six stages:

### IM stage 1: Needs assessment

This will establish the rationale for an occupational advice intervention within the population of interest by evaluating the discrepancy between current and desired practice. It will be achieved by combining information gathered using the following mixed methods approach:

- **Rapid evidence synthesis:** This will establish the published evidence relating to occupational advice interventions and examine the return to work measures used for outcome assessment. A database search of MEDLINE, EMBASE, CINAHL, CENTRAL, Cochrane Database of Systematic Reviews and Database of Reviews of Effectiveness will be undertaken for articles and systematic reviews relating to occupational advice interventions in patients undergoing elective surgical procedures published in the English-language in the last 20 years. PROSPERO registration number (CRD42016045235)

b. **Prospective cohort study:** The cohort study will collect information about how patients undergoing hip and knee replacement return to work following surgery, what interventions are currently being used, and how specific job demands influence workplace disability and productivity prior to and following surgery. The cohort study will be undertaken in 4 centres over a period of approximately 3-months. It will identify eligible patients who are a) aged over 16 years b) undergoing hip and knee replacement and c) in work in the 6 months prior to joint replacement. Exclusion criteria include: patients with a lack of mental capacity to understand and participate in the cohort study; patients who do not understand written and spoken English; emergency surgical procedures e.g. surgery for an indication of trauma; surgery performed for cancer or infection.

A minimum of 150 patients will be recruited (including a minimum of 60 hip and 60 knee replacements). Each patient will be assessed at baseline (peri-operatively) and at 8 and 16 weeks post-surgery. A subset of 45 patients will also be assessed at 24 weeks post-surgery. Collection of information about each participant at 3 or 4 time points will allow early functional recovery and return to work following surgery to be mapped. Baseline data collection will include patient demographic data and relevant occupational information. Assessment of functional status using joint specific: e.g. Oxford hip / knee score (OHS/OKS), Health utility: e.g. Euroqol (EQ5D-5 L), Patient health questionnaire (PHQ – 9), Brief Resilience Scale (BRS), Generalised anxiety disorder scale (GAD-2) and workplace measures: Workplace Limitations Questionnaire (WLQ) and elements of the Workplace Design Questionnaire (WDQ) will also be made. Follow up data collection will assess timing and manner of return to work (adaptions, phased return, amended duties) and usual activities, use of fit notes, healthcare utilisation, occupational advice received, in additional to repeated assessment of the functional outcomes performed at baseline.

The sample of 150 patients for the cohort study will be sufficient for representative estimates within an 8% margin of error with associated 95% confidence level [11]. Based on the rule of thumb of ten events per variable in logistic and cox regression, a sample size of 150 will allow a maximum of seven predictor variables to be included in the regression analyses; depending on the number of patients with the outcomes of interest (e.g. early return to work).

Quantitative data, derived from the cohort study questionnaires, will be analysed at York Trials Unit. Analyses will be undertaken in Stata. For each centre, current practice will be summarised including timing, content and delivery of current care pathways for hip and knee replacement patients and whether any additional interventions are provided for patients intending to return to work following surgery. Preoperative patient characteristics, and postoperative data will also be summarised. Logistic regression will be used to predict early return to work (within 6 weeks) including preoperative, operative and postoperative characteristics. In addition, a Cox proportional hazards model will be used to predict time to return to work in days from the date of the operation using the same covariates as the logistic regression.

c. **Patient and stakeholder interviews/focus groups:** Qualitative data will be collected from patients and other stakeholders (surgeons, general practitioners (GPs), employers, allied health professionals and nurses) at each study site. The purpose of these interviews is to obtain additional information about the shortcomings and difficulties with current care and support, how these might be overcome and how this might be translated in to an occupational advice intervention. From within the patient cohort, a subset of patients from a variety of work roles will be approached and invited to participate in interviews/focus groups. A purposive sample of between 10 and 15 patients in each centre (or a maximum of 45 patients from all centres) will be interviewed. Interviews will be conducted at 16 weeks post surgery to coincide with the follow up time points of the cohort study. This number of interviews was chosen as it should provide sufficient diversity of views and experiences to facilitate saturation of the thematic analysis.

Individuals from other stakeholder groups will also be interviewed. A total of 24 representatives from public, private, third sector, service and manufacturing employers including large organisations, small and medium-sized enterprises will be sampled. Interviews will also be conducted with orthopaedic surgeons (12 participants), occupational therapists, physiotherapists and nurses (12 participants), and GPs involved in the pre- and/or post-operative care of patients undergoing hip or knee replacement (12 participants).

A semi-structured interview method will be used to complete the interviews. Interviews will be digitally recorded and transcribed verbatim. Qualitative data, derived from the structured interviews, will be analysed thematically using the Framework Method [12]. This method is widely used in health research and particularly recommended for use in multi-disciplinary health research teams [13]. Following familiarisation with the data, the first few transcripts in each group will be independently coded by the interviewers, who will then compare, revise and agree a set of codes and/or categories to form a working analytical framework. This framework will be used to code the remaining transcripts in each group, but will remain flexible should further codes be identified. Summarised data will then be charted into a framework matrix to facilitate comparison of data across cases and groups as well as codes and categories. Potential themes will initially be identified independently by the interviewers who will then meet to discuss, revise and agree the final themes.

### IM stage 2: Identification of intended outcomes and performance objectives

Using the findings of Stage 1, the research team will specify who and/or what needs to change in order for workers to make a successful return to work following hip/knee replacement. A matrix of performance objectives for each stakeholder group will be constructed. The IM approach acknowledges that a number of factors might determine whether or not the performance objective is reached by considering both personal and external determinants of success.

### IM stage 3: Selection of theory-based methods and practical strategies

During this stage a list of possible intervention components matched to each performance objective/determinant will be generated, using theory, evidence, experience and consensus. As well as specific intervention components the most practical ways to implement these interventions will be identified.

### IM stage 4: Development of intervention components

The information and associated occupational advice strategies identified in the first three stages will be translated into specific tailored tools and materials to be considered as components for inclusion in our occupational advice intervention. A modified three-round Delphi process including all identified components will then be used to identify the strengths and weaknesses of these individual components and reach a final consensus on intervention content.

Using the modified Delphi process we intend to present information about potential components of the occupational advice intervention to the key stakeholder groups identified previously in order to seek their opinion and judgement on the likely content of the final intervention. To ensure wide participation and the validity of the consensus process we will recruit a minimum of 5 individuals from each stakeholder group. A maximum limit of 15 individuals from any given stakeholder group will be used to ensure one group’s opinions do not overwhelm the opinions of others within the consensus process. The proposed consensus process will involve a three round email based Delphi survey to all recruited stakeholders. We will follow the recommendations for reporting, developed by Sinha et al. [14] which focused on use of Delphi for development of core outcome sets, but which is applicable to the use of Delphi for other purposes. The initial questionnaire will be structured, including all identified components, asking panellists to rate items using a 4-level agreement scale (Strongly agree / agree / disagree / don’t know). An open-ended question will also be included to solicit additional suggestions about the intervention content. Round two questionnaires will include controlled feedback presenting modal round one rating for each item; reminding participants of their own previous ratings; and giving them the opportunity to change their ratings should they wish to do so. A consensus threshold of 70% will be pre-defined for analysis, similar to other previous studies [15, 16]. Consensus for this study will be defined as ≥70% ‘agree’ or ‘strongly agree’; or ≥ 70% ‘disagree’. The third round questionnaires will list the items that meet or exceed the consensus threshold and panellists will be asked to rank them in order of importance. The final ranked list will be used to develop the intervention.

The aim of this process will be to reach a consensus on:

1. The content of the occupational advice intervention using the components developed as part of Phase 1 and invited additional content from stakeholders within the first round of the Delphi process.
2. The favoured format, timing and method of delivery of the occupational advice intervention.
3. The essential qualities of a ‘return to work’ outcome measure based on previous collected information from IM stage 1.

Once consensus has been reached the research team will draft all of the ‘included’ components of the occupational advice intervention into a document and circulate it to Delphi panel members for final comment. Strategies for the delivery of the occupational advice intervention will be developed. These will be based on consensus information about the timing and mode of delivery (For example: paper based manual, electronic manual, supplementary online content). Finally a suitable ‘return to work’ outcome measure for use within the feasibility assessment will be defined. The process will conclude with a one-day meeting of the research team and steering group to finalise intervention content and design.

### IM stage 5: Development of an adoption and implementation plan

This stage focuses on the implementation and adoption of the intervention and will run concurrently with the final stages of intervention development as the content, format and method of delivery becomes finalised. The implementation plan will focus on intervention delivery in each of the study centres. It will be designed to address the gaps and/or barriers identified within these centres in Phase 1. Within the delivery frameworks assigned to each centre the methods and strategies to achieve the necessary change in behaviour given the institutional context will be formulated. This is likely to involve education and training of relevant staff at each site in the delivery of the intervention, but may involve other issues such as the design of the clinical pathway, alterations to length of hospital stay, clinical documentation, and staff skill mix and allocation etc. Appropriate support systems will be developed and an implementation plan constructed to assist adoption of the new occupational advice intervention within each of the study centres.

### IM stage 6: Evaluation and feasibility testing

The final stage of the intervention mapping process will focus on evaluating the practicality and acceptability of the intervention and its feasibility as a trial intervention. The feasibility stage will include not only an assessment of the intervention but also an assessment of the feasibility of undertaking a trial using the intervention. Delivery of the intervention is the key component in a future trial and as this is a newly developed intervention, testing the feasibility of delivery is crucial.

In this stage the cost of delivering the intervention will be also estimated; this will include type and grade of staff necessary to deliver the manual and the duration of these contacts. It will also assess the suitability of the intervention and selected ‘return to work’ measure as a future trial intervention and primary outcome measure respectively. The utility of the developed intervention as a tool for clinical practice will also be assessed alongside the evaluation of feasibility as a trial intervention.

The methods used to assess the intervention will be similar to those used in IM stage 1. In this stage we will recruit a total of 30 patients to undergo the intervention across the study sites. This group will be assessed using a questionnaire based return to work assessment supplemented by interviews with 15 of the recruited patients. Potential recruits will be identified using the following eligibility criteria a) aged over 16 years b) on the waiting list for hip or knee replacement c) in work prior to joint replacement and d) intending to return to work following surgery. The same exclusion criteria will be applied as used in IM stage 1.

This group will be assessed using a questionnaire based return to work assessment supplemented by review of all completed intervention paperwork. Stakeholder interviews will also be performed to gain a wider perspective on acceptability, practicality and utility of the new intervention. Using the methods described we will also collect and monitor other key information including a) patients’ and surgeons’ views on their willingness to participate in a future trial b) potential rates of recruitment and proportion of eligible patients consenting c) information about the behaviour and distributional characteristics of the selected ‘return to work’ outcome measures that will help inform the power calculation for any subsequent trial. This will allow us to make a recommendation about the feasibility of any subsequent trial.

**Fig. 1:**
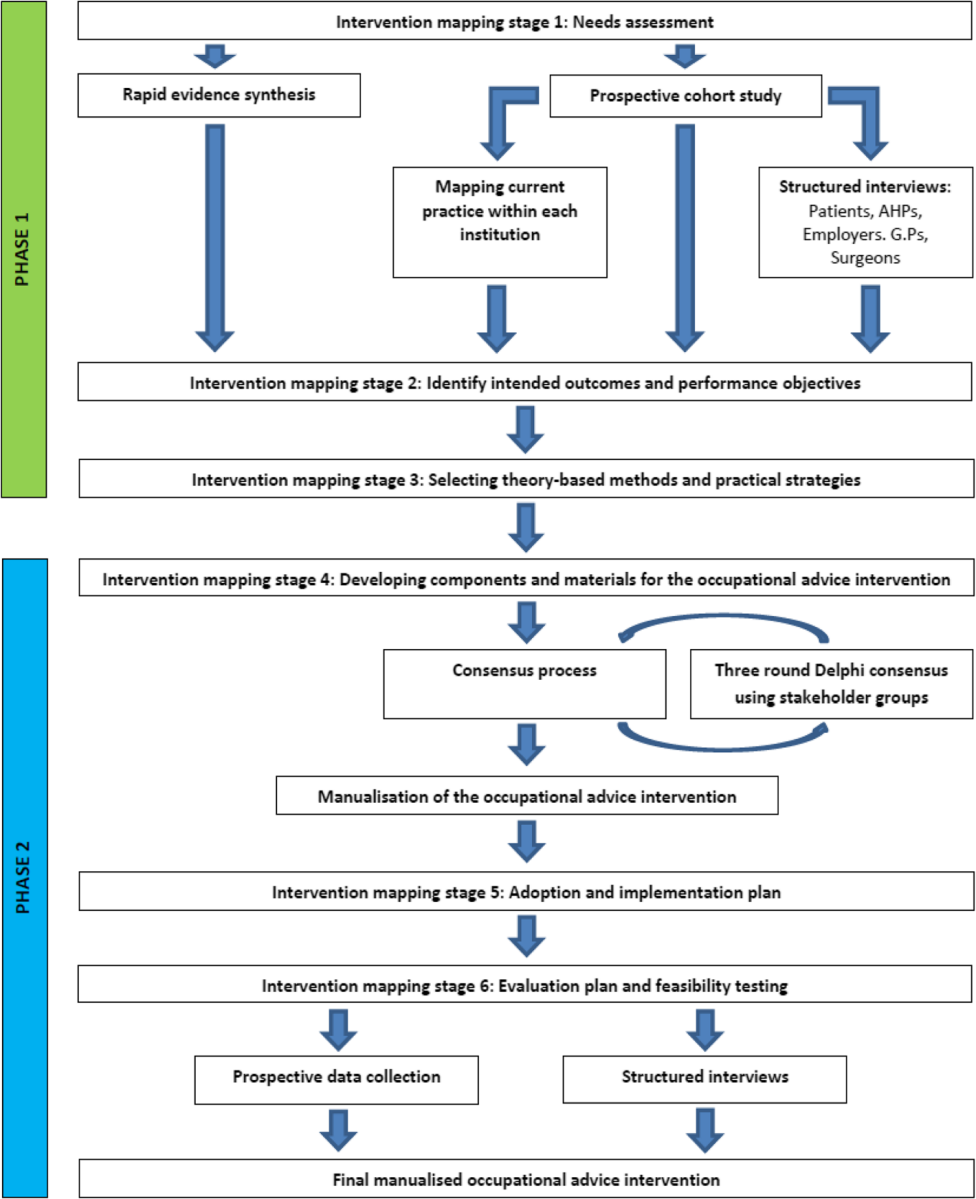
Diagrammatic overview of OPAL

The first 3 stages will be undertaken in Phase 1 with the final 3 stages undertaken in Phase 2 (Fig. 1).

## Discussion

The OPAL study is projected to take 27 months to complete. It commenced on the 1st July 2016 and is scheduled to end on the 1st October 2018.

### Project Management

The South Tees Hospitals National Health Service (NHS) Foundation Trust is the sponsor organisation for OPAL. Mr. Paul Baker is the chief investigator with overall responsibility for study conduct. The research team at South Tees NHS Trust are leading on the cohort study and the Delphi process. Researchers from Nottingham University are leading the qualitative interviews and co-ordinating the intervention development using the intervention mapping framework described previously. Researchers based at York University are undertaking the rapid evidence synthesis and providing methodological, statistical and health economic support. Study investigators meetings are held every 6–8 weeks to ensure the project is progressing as planned. The independent trial steering committee has oversight for the entire project and meets every 6–9 months at key intervals throughout the project.

## Abbreviations

BRS: Brief Resilience Scale
EQ5D-5 L: Euroqol health utility score
GAD-2: Generalised anxiety disorder scale
GP: General Practitioner
HRA: Health Research Authority
HTA: Health Technology Assessment
IM: Intervention Mapping
ISRCTN: International Standard Randomised Controlled Trials Number
NHS: National Health Service
NIHR: National Institute for Health Research
OHS: Oxford hip score
OKS: Oxford Knee Score
OPAL: Occupational advice for Patients undergoing Arthroplasty of the Lower limb
PHQ-9: Patient health questionnaire
PROSPERO: International prospective register of systematic reviews
WDQ: Workplace Design Questionnaire
WLQ: Workplace Limitations Questionnaire
